# Influence of right coronary artery motion, flow pulsatility and non-Newtonian rheology on wall shear stress metrics

**DOI:** 10.3389/fbioe.2022.962687

**Published:** 2022-08-09

**Authors:** Pratik Kandangwa, Ryo Torii, Peter D. Gatehouse, Spencer J. Sherwin, Peter D. Weinberg

**Affiliations:** ^1^ Department of Bioengineering, London, United Kingdom; ^2^ Department of Aeronautics, Imperial College London, London, United Kingdom; ^3^ Department of Mechanical Engineering, University College London, London, United Kingdom; ^4^ CMR Unit, Royal Brompton Hospital, London, United Kingdom

**Keywords:** coronary artery disease, computational fluid dynamics, transWSS, OSI, vorticity, Dean vortex, shear thinning

## Abstract

The patchy distribution of atherosclerosis within the arterial system is consistent with a controlling influence of hemodynamic wall shear stress (WSS). Patterns of low, oscillatory and transverse WSS have been invoked to explain the distribution of disease in the aorta. Disease of coronary arteries has greater clinical importance but blood flow in these vessels may be complicated by their movement during the cardiac cycle. Previous studies have shown that time average WSS is little affected by the dynamic geometry, and that oscillatory shear is influenced more. Here we additionally investigate effects on transverse WSS. We also investigate the influence of non-Newtonian blood rheology as it can influence vortical structure, on which transverse WSS depends; Carreau-Yasuda models were used. WSS metrics were derived from numerical simulations of blood flow in a model of a moving right coronary artery which, together with a subject-specific inflow waveform, was obtained by MR imaging of a healthy human subject in a previous study. The results confirmed that time average WSS was little affected by dynamic motion and that oscillatory WSS was more affected. They additionally showed that transverse WSS and its non-dimensional analogue, the Cross Flow Index, were affected still further. This appeared to reflect time-varying vortical structures caused by the changes in curvature. The influence of non-Newtonian rheology was significant with some physiologically realistic parameter values, and hence may be important in certain subjects. Dynamic geometry and non-Newtonian rheology should be incorporated into models designed to produce maps of transverse WSS in coronary arteries.

## Introduction

The highly non-uniform distribution of atherosclerosis within the arterial tree (Mitchell and Schwartz, 1965; Grøttum et al., 1983; Friedman et al., 1993) implies the existence of powerful local risk factors. Excessive uptake of plasma macromolecules by the arterial wall is one such factor; it shows excellent spatial correlation with rabbit and human lesions (Sinzinger et al., 1980; Sebkhi and Weinberg, 1994; Barnes and Weinberg, 1998; Weinberg, 2004). Another local factor – haemodynamically induced mechanical stress on endothelial cells – is likely to trigger the elevated uptake but the nature of the forces involved has been a matter of debate for many years.

The low, oscillatory wall shear stress (WSS) theory postulates that disease is triggered where haemodynamic WSS is low on average (Caro et al., 1971) and the Oscillatory Shear Index (OSI) is high (Ku et al., 1985). The OSI captures near-wall flow that deviates from the mean flow direction during the cardiac cycle (Ku et al., 1985). A more recent metric of multidirectional flow is the transverse WSS (transWSS), which captures components of instantaneous flow vectors that are perpendicular to the mean flow vector (Peiffer et al., 2013). Unlike the OSI, it does not include simple reverse flow. Its potential importance is predicated on the assumptions that endothelial cells elongate and align close to the mean flow direction and are adversely affected by flow across their long axis. TransWSS correlates better than low WSS or the OSI with patterns of permeability and lesions around side branches of the aorta (Mohamied et al., 2015). Furthermore, *in vitro* studies have shown that transverse flow leads to endothelial dysfunction (Wang et al., 2013; Ghim et al., 2018) and enhanced wall permeability (Ghim et al., 2017; Ghim et al., 2021), and that endothelial cells have evolved mechanisms which minimise it (Arshad et al., 2021).

Coronary artery disease has greater clinical importance than disease of the aorta. Conventional WSS metrics have been computed in static coronary models for over 20 years (e.g., Gibson et al., 1993; He and Ku, 1996; Perktold et al., 1998). TransWSS has also been investigated in such models: recent prospective studies have not demonstrated a convincing difference in plaque progression between regions experiencing low, intermediate or high transWSS (Kok et al., 2019; Hoogendoorn et al., 2020). However, the coronaries have a more dynamic mechanical environment than the aorta: there is greater bending, twisting and translation during the cardiac cycle. Only a few studies of coronary haemodynamics have included the effect of arterial motion.

Using a simple curved-tube model with a cyclically changing radius of curvature, Moore et al. (2001) found that WSS magnitudes could fluctuate substantially at individual locations in the tube but that flow patterns were broadly similar to those observed in stationary tubes and that the mean value was also predicted well by the stationary model. Zeng et al. (2003) imposed physiologically realistic motion on a constant-diameter model with an anatomically correct centre-line geometry and reached a similar conclusion: motion caused local fluctuations in WSS but the average value was similar to the static case. The fluctuations were in any case smaller than those produced by a pulsatile inflow boundary condition. Ramaswamy et al. (2004) incorporated cyclical diameter changes as well as motion of the vessel and their anatomically accurate geometry contained a stenosis. A notable finding was a substantial effect of motion on the pattern of OSI and on its average magnitude, which increased. Consistent with this, Torii et al. (2010) found that motion had a greater effect on OSI than on TAWSS in a dynamically and anatomically accurate model of a healthy right coronary artery (RCA).

The effect of arterial motion on transWSS has not previously been examined. The Dean-like secondary flow features that largely determine transWSS (Mohamied et al., 2017) are particularly sensitive to the changes in local curvature produced by coronary bending (Myers et al., 2001). The present study examines the effects of cyclical motion on WSS metrics including the transWSS in an anatomically realistic model of the normal human RCA. It also examines influences of blood rheology. Three previous studies of flow in coronary arteries found little difference in WSS patterns when non-Newtonian rheology was incorporated into the model (Johnston et al., 2004; Johnston et al., 2006; Soulis et al., 2006), but Apostolidis et al. (2016) showed differences as high as 50% at the left coronary bifurcation. A study by Cherry and Eaton showed that errors arising from the assumption of Newtonian rheology were particularly high in the presence of secondary flows (Cherry and Eaton, 2013). Indeed, non-Newtonian rheology can lead to the appearance of additional vortices in models of curved arteries (van Wyk et al., 2015). Hence shear-thinning properties might be particularly critical for transWSS. The present study examines the effect of non-Newtonian rheology on patterns of transWSS under coronary artery motion.

## Methods

### Data acquisition using magnetic resonance imaging

The dynamic geometry used in this analysis was derived from the 4-D MRI data of Torii et al. (2010). Fluid-structure interaction (FSI) techniques, used for other vessels, are not appropriate because most of the deformation in the coronary arteries is caused by cardiac motion and not by the fluid within the artery itself. An additional advantage of using MRI is that flow waveforms can be obtained at the same time, with the same imaging modality.

Briefly, data for the right coronary artery (RCA) of a healthy 31-year-old male were acquired using an ECG- and navigator-gated interleaved spiral sequence on a Siemens Sonata 1.5 T scanner. Written consent was obtained from the volunteer, and the protocol was approved by the local ethics committee; the study complied with the Declaration of Helsinki. A total of 20 interleaves were required to fill the k-space, with each lasting 10 ms. Imaging was performed during free-breathing and a water excitation pulse was incorporated to eliminate signal from fat. The spatial resolution of the acquired images was 0.9 × 0.9 mm; the images were later reconstructed to a finer resolution of 0.45 × 0.45 mm. Approximately 15–21 cross sections were obtained along the length of the RCA at 14 different time points. Segmented lumen contours from the cross sections were interpolated using cubic splines for 3-D surface reconstruction. The cross-sectional shape of the reconstructed geometry was assumed to be constant throughout the entire cardiac cycle as effects of radial changes are small (Torii et al., 2010).

**FIGURE 1:**
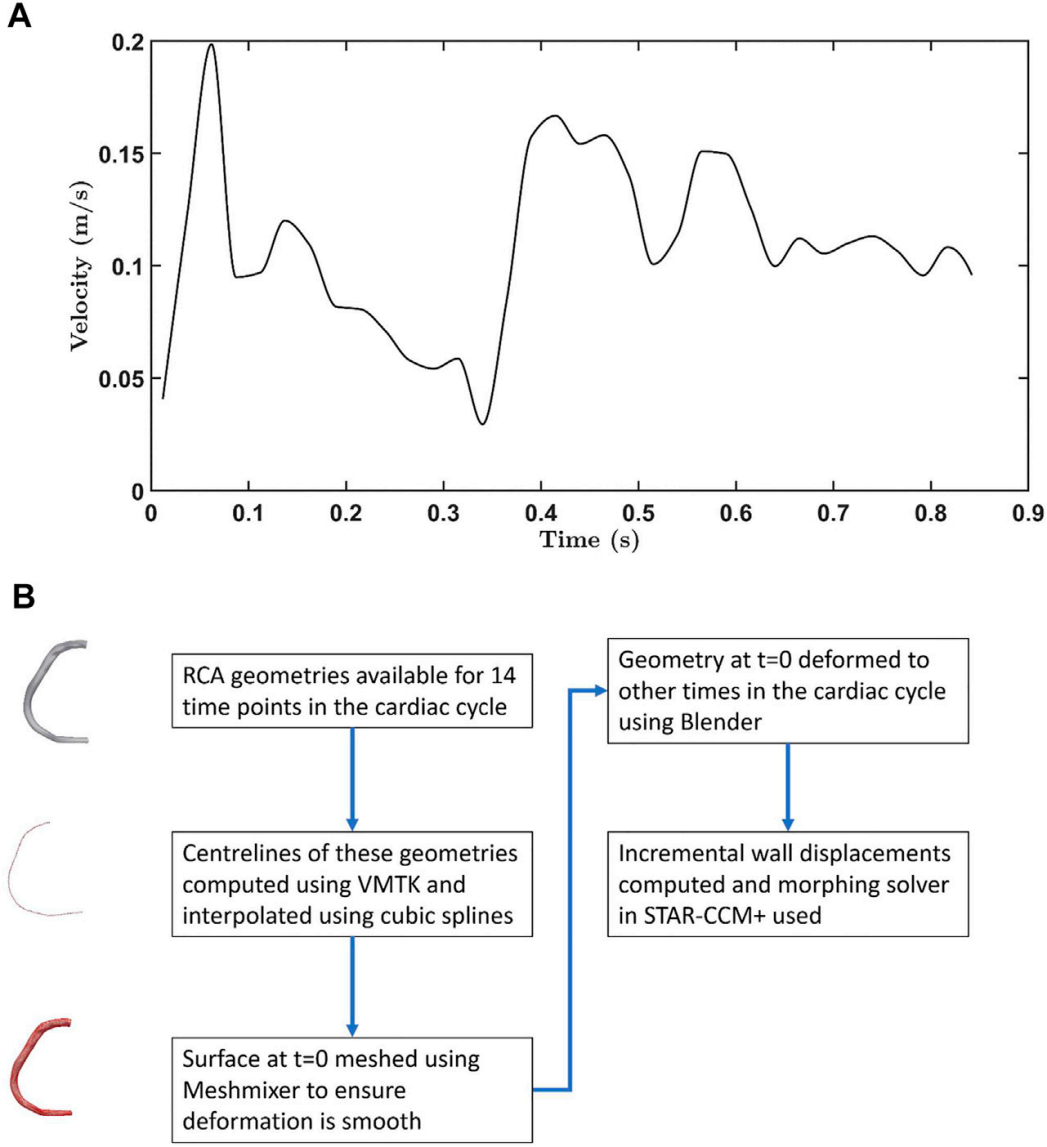
**(A)** Velocity waveform for the RCA. Data were obtained from 0.012 s onwards. Earlier values were obtained by extrapolation, using a spline. **(B)** Flowchart showing how the moving wall boundary condition was generated for flow simulations.

An ECG- and navigator-gated interleaved spiral phase velocity mapping sequence (Keegan et al., 2004) was used to obtain velocity data in the proximal region of the RCA in the same volunteer. Figure 1A shows how the measured velocity varied throughout the cardiac cycle. The phases of geometry and velocity data were aligned using the timing of the R-wave (i.e. the beginning of the cycle), time from the R-wave and the RR interval as references.

### Computational framework

Numerical methods were used to model blood flow in the coronary artery. A finite volume approach implemented in STAR-CCM+ (Siemens Industry Software Ltd., UK) approximated the solution of the continuity and Navier-Stokes equations. More specifically, a segregated flow solver was used to solve the equations in an uncoupled manner using a predictor-corrector scheme. A second order upwind scheme was employed for spatial discretisation and a first order Euler implicit scheme for temporal discretisation. Details of how the transient, convective, diffusive and pressure terms in the Navier-Stokes equation are discretised can be found in the STAR-CCM user guide. Polyhedral meshing was used because of its shorter computational time and greater wall shear stress accuracy (Spiegel et al., 2011); eight prism layers were added to capture the effect of the boundary layer near the wall. The flow was assumed to be laminar, and blood was assumed to be incompressible (with density 1,044 kg/m^3^) and homogeneous.

The flow waveform shown in Figure 1A and a flat velocity profile (i.e. uniform through-plane velocity) were prescribed at the inlet, and a zero relative pressure condition at the outlet. When the inlet and outlet were extended by 10 vessel diameters in trial computations, the results did not change substantially: the Spearman rank correlation coefficient for transverse wall shear stress between the case with and without extensions was 0.98. However, the morphing method (described below) did not work well near the proximal root and required a slight inlet extension to ensure smooth deformation. Hence, all simulations were performed with inlet extensions having a length equal to the vessel diameter.

The boundary condition for the moving wall was incorporated using a combination of the Vascular Modelling Toolkit (VMTK), Meshmixer, Blender and the mesh morphing solver in STAR-CCM+. Figure 1B summarises the method used to generate wall displacements. First, the centrelines were computed for the RCA geometries acquired at 14 timepoints, using VMTK. Interpolation using cubic splines gave centrelines at intermediate time intervals. Surface deformation in Blender was implemented by using centrelines (50 control points) of geometries as source and target points, and involved three steps: (i) rigging, (ii) binding, and (iii) skinning. During the rigging phase, the armature or bone structure of the 3-D RCA geometry was created using its centreline. Bones were then bound to the mesh using the bone heat algorithm (Baran and Popović, 2007) in which each mesh vertex is assigned a weight based on its distance from a particular bone. The weight controls how much influence the movement of the bone has on the vertex. The RCA was deformed during the skinning phase using linear blend skinning, also known as skeleton subspace deformation (Lewis et al., 2000). Finally, incremental wall displacements were computed for each time step; when combined with the morphing algorithm in STAR-CCM+, this allowed non-rigid deformations of the mesh by redistributing mesh vertices. Deformation of an exemplar geometry throughout the cardiac cycle is shown in Supplementary Figure S1, and values of curvature and torsion along the RCA are given in Supplementary Figure S2. A comparison of the original images and derived geometries in ParaView showed only negligible differences (data not shown).

To validate the methods, flow was computed in a 180-degree curved tube with sinusoidally varying curvature and steady inlet velocity, as modelled by Santamarina et al. (1998). Sensitivity tests were carried out to determine the optimal time step, mesh resolution and number of cardiac cycles.

### Wall shear stress metrics

Wall shear stress vectors from the simulation were used to compute the four metrics defined in Equations (1)–(4). Time average WSS (TAWSS) is a magnitude-based metric and not sensitive to flow direction. OSI (the equation in (He and Ku, 1996) was used) and transWSS (Peiffer et al., 2013) have been described above. The Cross Flow Index (CFI) is similar to transWSS in that it represents the directionality of WSS, but it does not take magnitude into account. Mathematically it is the average over the cardiac cycle of the sine of the angle between the temporal mean WSS vector and instantaneous WSS vector (Mohamied et al., 2017).

$$Time\;Averaged\;Wall\;Shear\;Stress\;\left(TAWSS\right)=\frac{1}{t}\overset{T}{\underset{0}{\int }}\left|\vec{\tau_{w}}\right|dt$$ (1)

$$Oscillatory\;Shear\;Index\;\left(OSI\right)=\frac{1}{2}\left(1-\frac{\left|\int_{0}^{T}\vec{\tau_{w}}dt\right|}{\int_{0}^{T}\left|\vec{\tau_{w}}\right|dt}\right)$$ (2)

$$Cross\;Flow\;Index\;\left(CFI\right)=\frac{1}{T}\overset{T}{\underset{0}{\int }}\frac{\vec{\tau_{w}}}{\left|\vec{\tau_{w}}\right|}\cdot \left(\vec{n}\times \frac{\int_{0}^{T}\vec{\tau_{w}}dt}{\left|\int_{0}^{T}\vec{\tau_{w}}dt\right|}\right)dt$$ (3)

$$Transverse\;Wall\;Shear\;Stress\;\left(transWSS\right)=\frac{1}{T}\overset{T}{\underset{0}{\int }}\left|\vec{\tau_{w}}\cdot \left(\vec{n}\times \frac{\int_{0}^{T}\vec{\tau_{w}}dt}{\left|\int_{0}^{T}\vec{\tau_{w}}dt\right|}\right)\right|dt$$ (4)

where $\vec{\tau_{w}}$ is the instantaneous WSS vector, $\vec{n}$ is the surface normal and T is the period of the cardiac cycle.

In order to investigate the effect of vessel motion on these metrics the following three simulations were undertaken:

i) Static Pulsatile – pulsatile velocity with static geometry, to isolate the unsteady effect of flow pulsatility. (The geometry was obtained at t = 0, defined as 50 ms after the R-wave of the ECG.)
ii) Dynamic Nonpulsatile – constant velocity at the inlet with wall movement, to isolate the unsteady effect of RCA motion. (The velocity was the mean over the cardiac cycle.)
iii) Dynamic Pulsatile – pulsatile velocity at the inlet with wall motion, to give the most physiologically realistic solution.

For (ii) and (iii), it is important that the moving reference frame is taken into account when computing OSI, CFI and transWSS. Since the vessel wall is moving and the mesh elements are constantly reorienting, the conventional postprocessing method of mapping WSS vectors at all time steps on to the t = 0 wall geometry results in out-of-plane vectors and can lead to errors. To avoid this, coordinate transformation was computed between t = 0 and the time under consideration for each surface element, and the vector was transformed appropriately.

Distributions of the shear metrics computed in the RCA geometry were converted into 2-D rectangular maps using the t = 0 anatomy as the reference geometry. Parametrisation implemented in VMTK used the curvilinear abscissa and the angular position of each point on the wall in a plane perpendicular to the local centreline. These longitudinal and circumferential coordinates of the rectangular parametric space were used to make the cut along the inner wall of the vessel (the inner curvature at t = 0) and subsequently open the surface using Python, as shown in Supplementary Figure S3. Spearman rank correlation coefficients were computed to quantify similarity between maps. To facilitate this, maps were divided into individual elements using a square grid of 19 × 19 µm (which is smaller than the mesh resolution); pixels were ranked according to their value of the metric being mapped.

### Non-Newtonian models

Non-Newtonian blood rheology was investigated using the generalised Carreau-Yasuda model. Eq. 5 defines the dependence of dynamic viscosity (µ) on shear rate ( $\dot{\gamma }$ ) in this model:


$$\mu =\mu_{\infty }+\left(\mu_{0}-\mu_{\infty }\right){\left[1+{\left(\lambda \dot{\gamma }\right)}^{a}\right]}^{\frac{n-1}{a}}$$ (5)

where $\mu_{0}$ is viscosity at zero shear rate, $\mu_{\infty }$ is viscosity at infinite shear rate and λ , *a* and *n* are Carreau-Yasuda rheological parameters (Mahmood et al., 2020).

The parameters are determined empirically and vary significantly with haematocrit, gender, cholesterol level and fitness index. Four different parameter sets (from Cho and Kensey (Cho and Kensey, 1991), Gijsen et al. (Gijsen et al., 1998), van de Vosse et al. (Van de Vosse et al., 2003) and Chen et al. (Chen et al., 2006)) were used (Supplementary Table S1).

To better understand the importance of non-Newtonian effects, we calculated local and averaged global non-Newtonian importance factors, as defined by Johnston et al. (Johnston et al., 2004) and Ballyk et al. (Ballyk et al., 1994), respectively. The local importance factor is simply the ratio of non-Newtonian viscosity to Newtonian viscosity (Eq. (6)) for each mesh point. Values significantly different from one would indicate a non-Newtonian regime. The global importance factor (Eq. (7)) is a spatially averaged measure of deviation from the Newtonian approximation. Values greater than 0.25 indicate non-Newtonian flow behaviour (Soulis et al., 2008; Soulis et al., 2011).

$$I_{L}=\frac{\mu }{\mu_{\infty }}$$ (6)

$$I_{G}=\frac{1}{N}\frac{{\left[\sum_{N}{\left(\mu -\mu_{\infty }\right)}^{2}\right]}^{\frac{1}{2}}}{\mu_{\infty }}\times 100$$ (7)

where *N* is the total number of mesh points on the surface.

## Results

### Validation and sensitivity analysis

**FIGURE 2:**
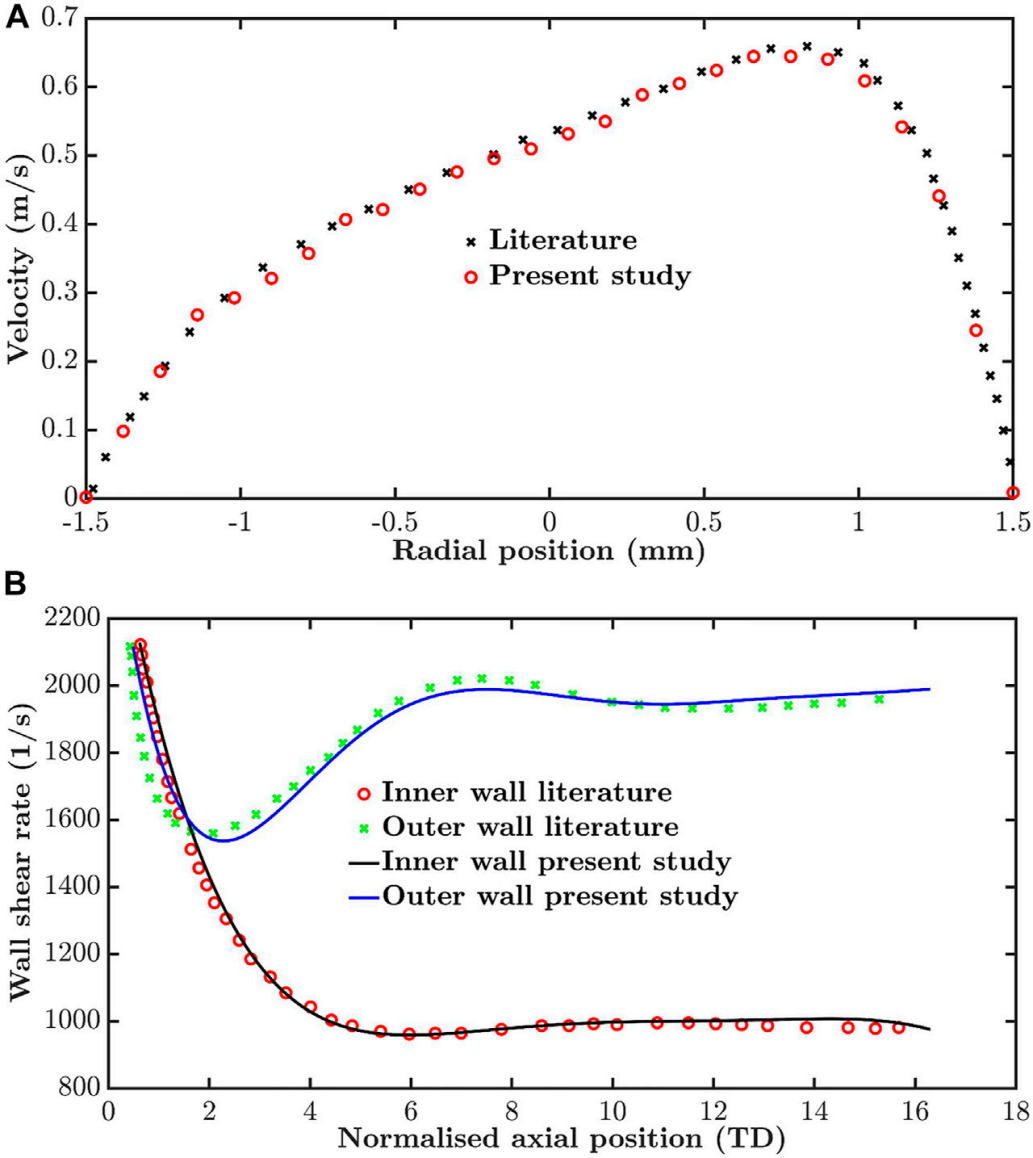
Validation by comparison of simulations for a tube of time-varying curvature with previously published values for the same dynamic geometry. **(A)** Velocity profiles thirteen tube diameters from the inlet and **(B)** wall shear rate along the inner and outer wall, both at t = 0.25 s in a period of 1 s.

The computational method, including mesh morphing, used to model the flow in the moving RCA was validated by using it to simulate flow in a tube with dynamic curvature, for which results obtained using experimentally validated numerical methods are already available (Santamarina et al., 1998). Figure 2A compares axial velocity profiles 13 tube diameters from the inlet at t = 0.25 s of a 1 Hz cycle: the average difference between velocity values was 3.5%. Figure 2B compares newly computed and previously reported wall shear rates along the inner and outer wall of the tube at the same time. The average differences were 1.2 and 1.6%, respectively.

**FIGURE 3:**
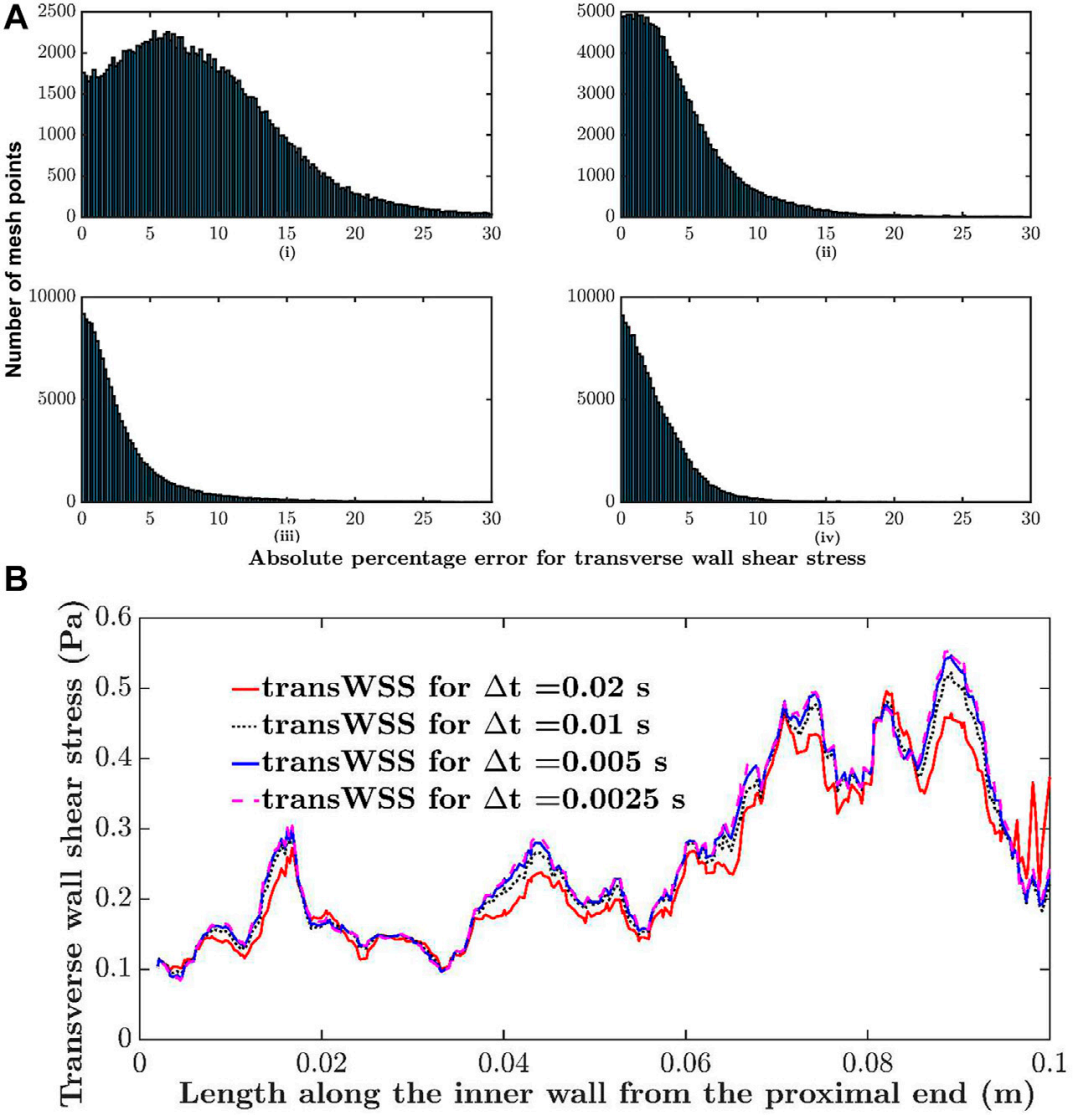
Sensitivity analysis. **(A)** Histogram comparing absolute percentage differences in transWSS at each mesh point between different mesh resolutions: i) 90 k elements vs. 278 k elements, ii) 278 k elements vs. 525 k elements, iii) 525 k elements vs. 626 k elements and iv) 626 k elements vs. 775 k elements. **(B)** Transverse wall shear stress along the inner wall of the RCA, computed with different time steps (∆t).

To assess the parameter values required to obtain convergence, simulations were carried out for different mesh resolutions, time steps and number of cardiac cycles until the average of the absolute difference between successive iterations across all mesh points and for all four metrics was approximately 2.5%. For example, the maximum base size was repeatedly halved (element count increased) until the criterion was satisfied. The histograms in Figure 3A show the difference in transWSS estimates between different mesh resolutions. Differences reached 30% at some mesh points when comparing coarse meshes (90 k elements vs. 278 k elements) but were below 10% for almost all the points when comparing the finer meshes (626 k elements vs. 775 k elements). Average absolute percentage differences for TAWSS, CFI, OSI and transWSS between the meshes with 626 k and 775 k elements were 1.3, 2.1, 2.4 and 2.6%, respectively. Therefore, 775 k elements was considered sufficiently fine for mesh independence.

A similar analysis showed that a time step size of 0.005 s and two cardiac cycles were sufficient; further halving of the time step or increasing the number of cycles did not change the results. For example, Figure 3B shows transWSS along the inner wall of the RCA for four different time step sizes. The change is minimal between 0.005 and 0.0025 s. WSS metrics were calculated over the two cycles.

### Influence of RCA motion and flow pulsatility on WSS metrics

**FIGURE 4:**
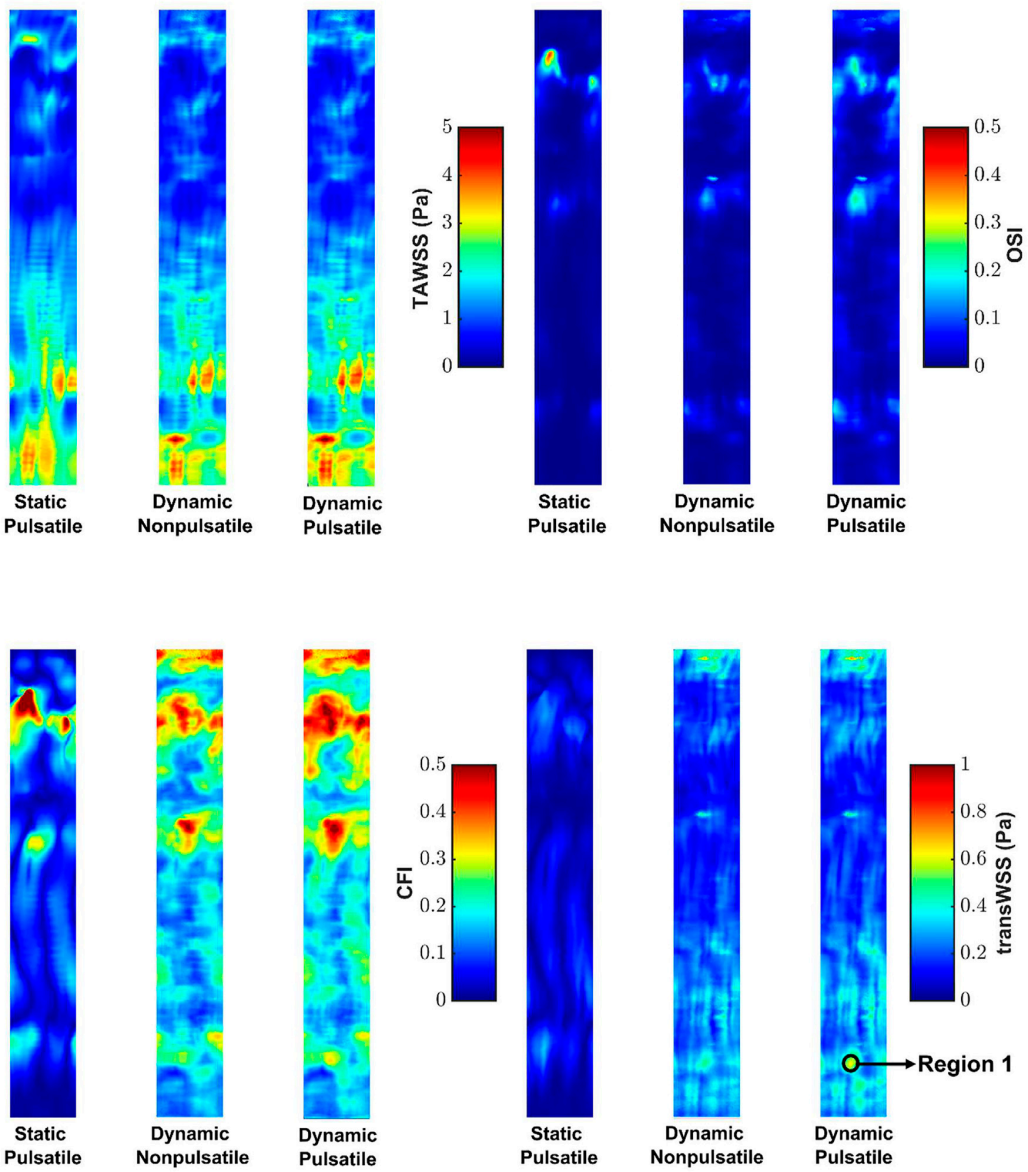
Maps of TAWSS, OSI, CFI and transWSS for the three cases. Mean RCA flow is from top to bottom.

Figure 4 shows maps of the four shear metrics for the three cases. Little change is visible in the patterns of TAWSS and OSI between cases, whereas substantial differences are evident in the patterns of CFI and transWSS. Table 1 gives the Spearman rank correlations between the three cases for each metric. Correlation coefficients show that wall motion did not alter TAWSS by much—the correlation coefficient between static pulsatile and dynamic pulsatile cases was 0.93—and that it had by far the largest effect on transWSS (coefficient of 0.19). Ignoring wall motion could lead to intermediate but still substantial differences for the other two metrics, as shown by their correlation coefficients of 0.58 (CFI) and 0.75 (OSI). The fact that transWSS and CFI gave lower coefficients than the OSI shows that wall motion affects truly multidirectional flow more than reversing flow. On the other hand, the consistently high coefficients (all ≥0.94) for the dynamic cases with and without flow pulsatility show that the unsteady effects of flow pulsatility are essentially negligible. Similar observations were made for maps of instantaneous WSS throughout the cardiac cycle: wall motion had a greater effect than pulsatility. Supplementary Table S2 summarises the Spearman rank correlation coefficients at different time points for WSS maps under the three conditions. Note that using rank correlation coefficients means that the patterns are compared but the magnitudes are not.

**TABLE 1:** Spearman rank correlation coefficient between different maps.

	Metric			
	TAWSS	OSI	CFI	transWSS
Static Pulsatile vs. Dynamic Pulsatile	0.93	0.75	0.58	0.19
Dynamic Nonpulsatile vs. Dynamic Pulsatile	0.99	0.94	0.94	0.96

### Influence of non-Newtonian rheology on RCA hemodynamics

Supplementary Figure S4 shows the evolution of the global importance factor over the cardiac cycle for the four published combinations of Carreau-Yasuda parameters in simulations with pulsatile flow and dynamic geometry. The threshold line identifies the level above which non-Newtonian effects are considered important. Non-Newtonian effects were important for two of the four models.

**FIGURE 5:**
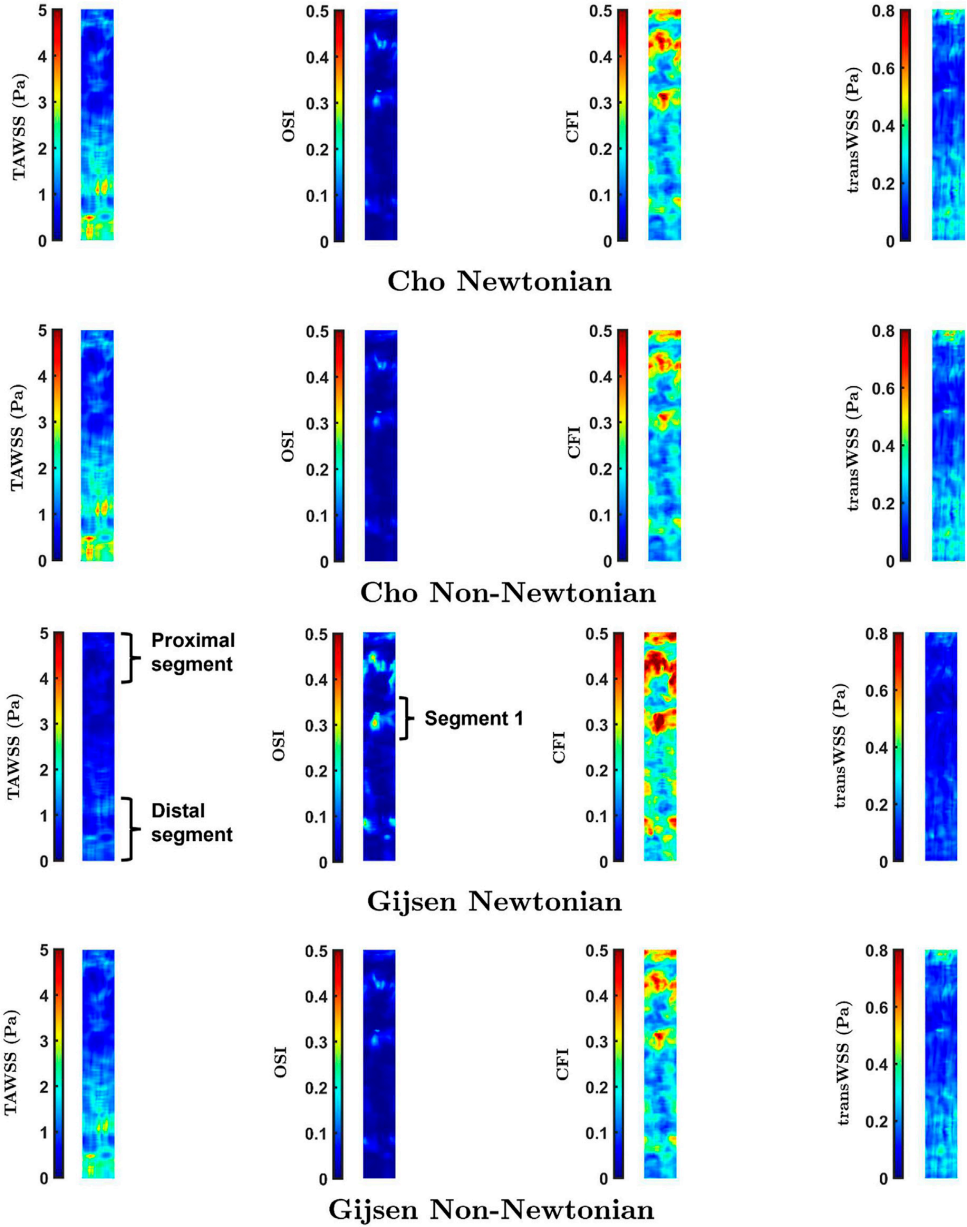
Maps of TAWSS, OSI, CFI and transWSS in the Dynamic Pulsatile case for Newtonian and non-Newtonian rheology using the models of Cho and Kensey (Cho and Kensey, 1991), and Gijsen et al. (Gijsen et al., 1998)

Figure 5 shows maps of the four shear metrics obtained from simulations of the dynamic pulsatile case using the Newtonian and non-Newtonian parameters of Cho and Kensey (Cho and Kensey, 1991) and Gijsen et al. (Gijsen et al., 1998). These are the most extreme cases: the global importance factor is consistently below the threshold line for the former whereas the latter has a global importance factor well over the threshold line throughout the cardiac cycle. As the parameters were determined empirically, the Newtonian viscosity is different for the two cases.

While the parameters of Cho and Kensey give Newtonian and non-Newtonian maps that are essentially identical, distinct differences are visible between Newtonian and non-Newtonian maps obtained with the Gijsen et al. parameters. For all four metrics, these differences are seen primarily in the proximal and distal segments of the vessel, labelled in the third row of Figure 5. Additionally, the maps of OSI and CFI show clear differences in the region labelled Segment 1. Spearman rank correlation coefficients between the Newtonian and non-Newtonian versions of the Cho and Kensey model were 0.99 for all metrics. Although the equivalent correlation coefficients were lower for the Gijsen et al. model they were still high, the lowest value being 0.95 for OSI. Despite these high rank correlations, introducing non-Newtonian rheology produced changes in pattern (as opposed to simple scaling effects) that are easily visible in all the maps in Figure 7.

## Discussion

The main aim of this study was to examine the effect of wall motion on a range of computed WSS metrics, including transWSS, in the RCA using fully subject-specific geometry, motion and inflow waveform. A secondary aim was to investigate the effect of non-Newtonian blood rheology in the model. It was predicted that metrics capturing near-wall flow components perpendicular to the mean vector – i.e, the CFI and transWSS – would be affected more than the traditional TAWSS and OSI by movement and non-Newtonian rheology as a result of their dependence on time-varying secondary flows. The geometric and flow boundary conditions were obtained in a young, healthy subject; the influence of stenotic disease on WSS metrics, and on the sensitivity of those metrics to motion and rheology, was therefore not considered. Preliminary work optimised the mesh resolution, time step and number of cardiac cycles that were modelled, and showed that the method gave results similar to those previously obtained in an idealised geometry comprising a regular tube with dynamic curvature.

The maps of WSS metrics and the Spearman rank correlation coefficients computed across the different cases showed that wall motion had minimal effect on TAWSS, which is consistent with earlier studies (Santamarina et al., 1998; Zeng et al., 2003; Torii et al., 2010) and theoretical expectations (Lynch et al., 1996). The OSI was found to be more sensitive than TAWSS to changing geometry during the cardiac cycle, as reported by Torii et al. (Torii et al., 2010). A novel finding was that still larger effects of wall motion were seen for the CFI and, even more so, for transWSS. Hence coronary motion should not be ignored when mapping these two metrics. The pulsatile nature of the inflow waveform had much smaller effects.

Polar plots were generated to further understand changes to the multidirectional nature of the flow. Such plots track the direction and magnitude of the instantaneous WSS vector over the cardiac cycle at a single location on the wall. Each point in the plot represents the position of the tip of a WSS vector with its origin at 0,0. The *X* and *Y* axes in the plot represent WSS components oriented along the circumference and axis of the vessel, respectively. For truly multidirectional flow of constant magnitude, the tip of the WSS vector would trace out a perfect circle. Uniaxial flow would appear as a straight line through the origin.

**FIGURE 6:**
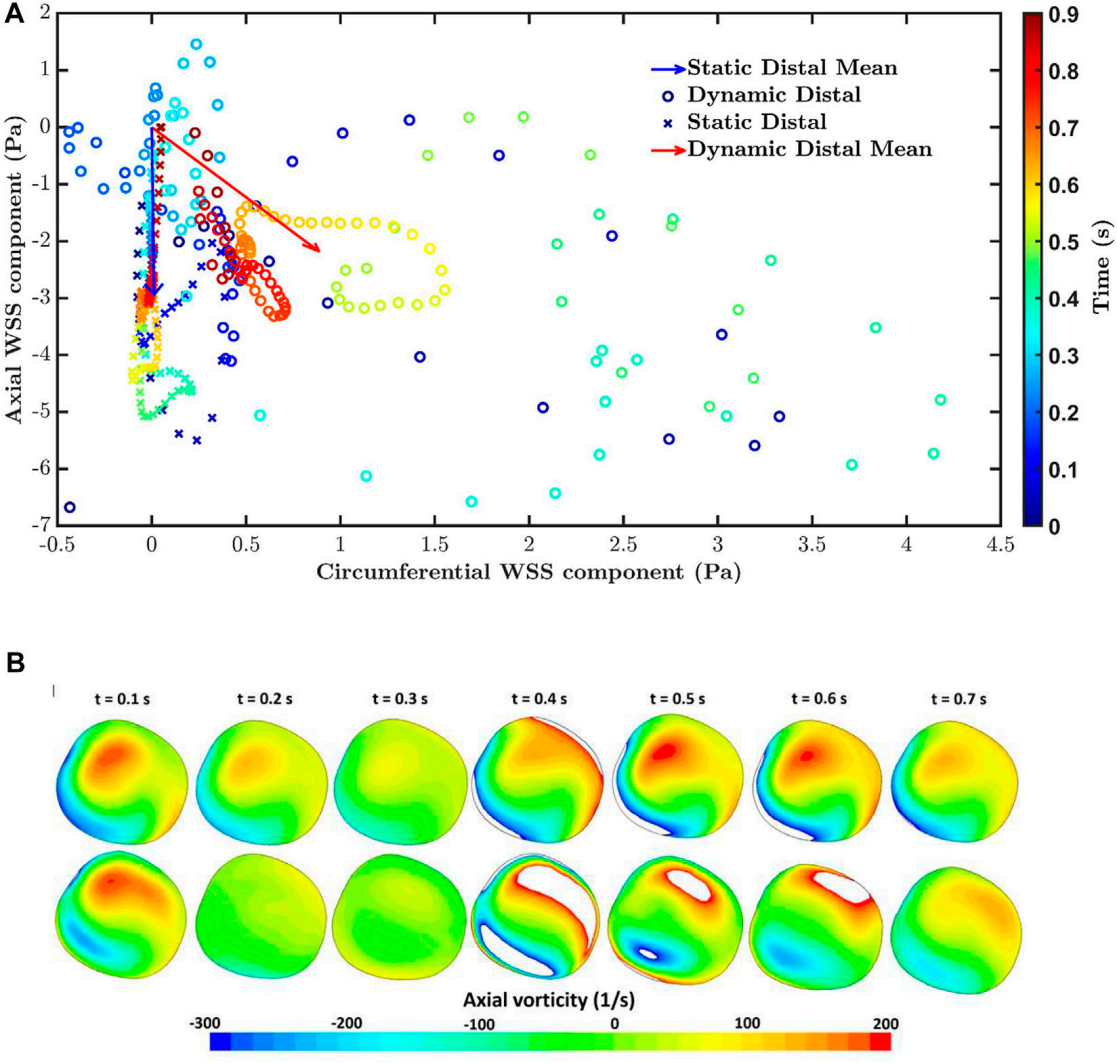
**(A)** The polar plot and mean vector for static pulsatile and dynamic pulsatile cases for a distal location in the RCA (Region 1 in Figure 4), where transWSS differed substantially between the two cases. Negative values of axial WSS indicate flow travelling down the vessel. **(B)** Axial vorticity for the static pulsatile case (top) and dynamic pulsatile case (bottom) across the RCA in region one at different times in the cardiac cycle. Positive and negative vorticity values beyond the range of the colour bar scale are shown as white.

Figure 6A shows the polar plot and the mean vector under static pulsatile and dynamic pulsatile conditions for a distal location in the RCA (Region 1 in Figure 4) that is far from boundaries and where transWSS differed substantially between the two cases. For the static pulsatile case, Figure 6A shows that instantaneous WSS vectors are aligned with the mean WSS vector, in the forward going axial direction, throughout most of the cardiac cycle. In the dynamic pulsatile case, on the other hand, the mean WSS vector – although of similar magnitude – has a significant circumferential component (exaggerated by the different x- and *y*-axis scaling in the figure) and the instantaneous WSS vectors show larger deviations from this mean direction, including in the reverse direction; the magnitudes are again not hugely different from the first case.

Figure 6B compares axial vorticity across the distal RCA, in Region 1, for the static pulsatile and dynamic pulsatile cases. Changes in vortical strength through the cardiac cycle are greater for the dynamic case than the static case. This is consistent with a previous analytical study showing that time-varying curvature can even reverse the direction of Dean vortices (Waters and Pedley, 1999). Furthermore, the static case consistently has a positive dominant vortex throughout the cardiac cycle, presumably due to the constant out-of-plane deformation of the RCA geometry (Caro et al., 1995), whereas two distinct vortices with more equal (albeit changing) strength are seen in the dynamic case, perhaps because the degree of non-planarity is changing. The relation of vorticity to CFI and transWSS is complex because the mean axial velocity also changes over the cardiac cycle. Nevertheless, the large differences in vortical strength and structure between the two cases presumably account for the particularly large differences in CFI and transWSS.

**FIGURE 7:**
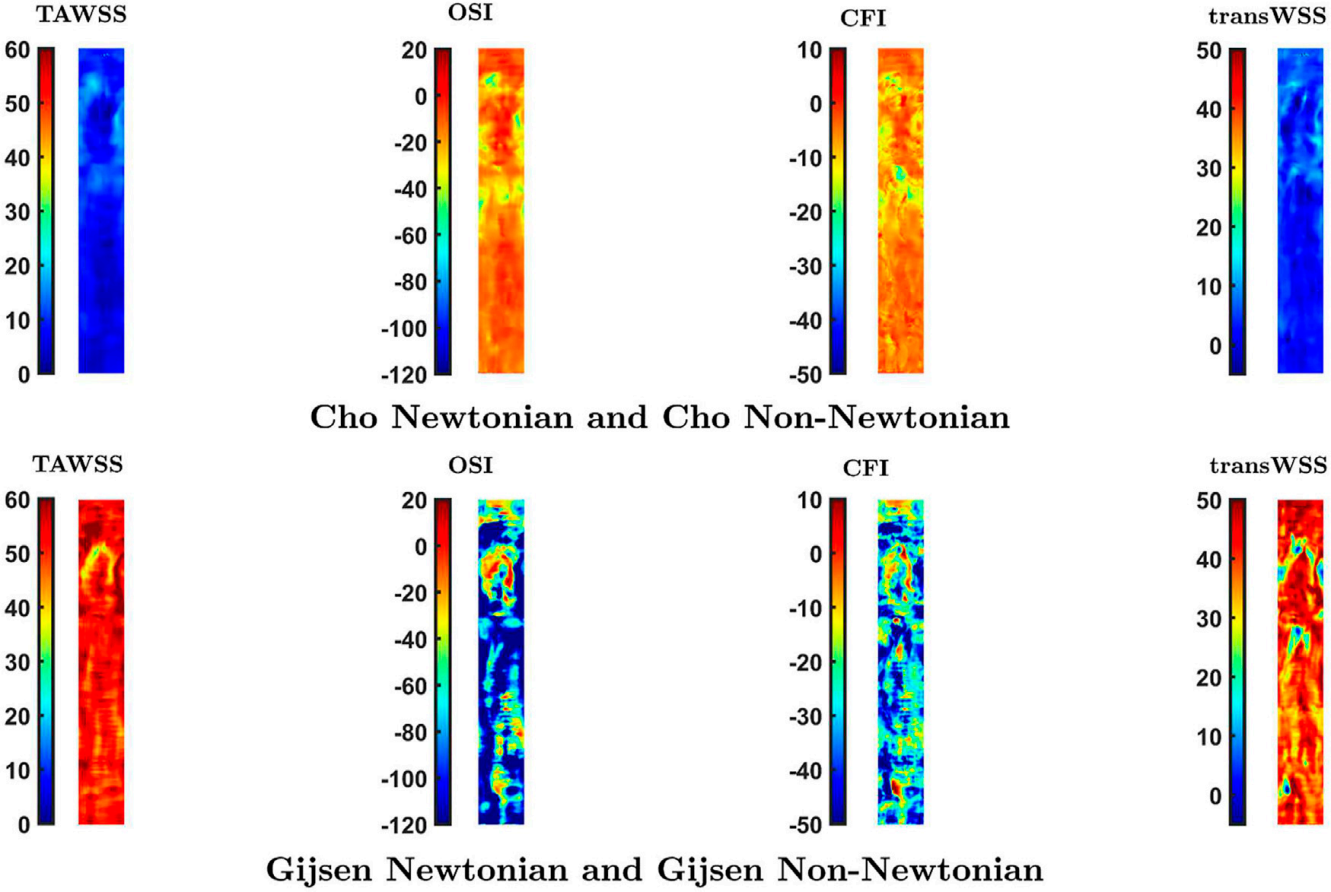
Percentage difference in values of the four WSS metrics at each location caused by a switch from a Newtonian to a non-Newtonian model. Note that the colour scale is the same for each vertical pair of maps.

Simulations carried out to study the effect of non-Newtonian blood rheology revealed a dependence on the choice of parameter values. These are determined empirically and may vary between subjects, implying that the effect of non-Newtonian rheology may also vary between subjects. There was a high Spearman rank correlation coefficient for all comparisons. Note, however, that magnitudes can be scaled in a non-linear way and still give a high correlation coefficient based on rank. To provide further information, Figure 7 shows maps of the percentage difference in WSS metrics between Newtonian and non-Newtonian rheology, for both the Cho and Kensey model (Cho and Kensey, 1991) and the model of Gijsen et al. (Gijsen et al., 1998). With the Cho and Kensey model, differences are minimal for all four metrics, as also seen in Figure 5, but that is not the case for the model of Gijsen et al. Not only are there large differences in magnitude between the Newtonian and non-Newtonian assumptions but there are large variations in the difference from point to point in the map, ranging from nearly 25% to approximately -250% for OSI, 15% to -80% for CFI and 60% to -5% for transWSS. Discrepancies for TAWSS are also high, but more uniform. Thus, this non-Newtonian model does appear to have an influence other than simple scaling on parameters related to the directionality of flow.

Supplementary Figure S5 shows how the non-Newtonian viscosity varies with shear rate for the two models. The non-Newtonian viscosity converges to Newtonian viscosity (viscosity at infinite shear) relatively quickly for the Cho and Kensey model. This means that the non-Newtonian effect comes into play only for a limited spatio-temporal window during the cardiac cycle, where and when shear rates are low. In the Gijsen et al. model, however, the non-Newtonian viscosity does not converge to the Newtonian value even for high values of shear rate, which means that the non-Newtonian effect is significant at most locations throughout the whole cardiac cycle. According to these parameter values, the Newtonian approximation underestimates the magnitude of the dimensional metrics, TAWSS and transWSS, most likely through a direct influence of the lower viscosity. However, the lower Newtonian viscosity overestimates the non-dimensional metrics, OSI and CFI, presumably because the lower viscosity means less damping of flow disturbances. This effect will also influence transWSS.

Finally, we separate effects of fluid moving past the wall from those of the wall moving past the fluid. As the vessel deforms, distances between points on the wall are altered. In the simplest case, if a cylinder is bent into a curved tube having the same centreline length, the inner curvature must get shorter and the outer curvature must get longer. Even in the absence of any imposed flow, there will be relative motion between the wall and fluid inside the cylinder, leading to the generation of WSS. This type of effect will occur in the RCA *in vivo*. We have investigated its magnitude by examining WSS metrics in a model with dynamic geometry but no inflow. The resulting map of TAWSS is given in Supplementary Figure S6. Averaged over the map, TAWSS was 28% of the value seen with the same dynamic geometry but a pulsatile inflow, in which shear is generated by wall movement *and* fluid movement. Because of the way the problem is posed (zero flow at the inlet, free flow at the outlet, inlet anchored in space), the TAWSS generated by wall motion increases with distance down the artery.

WSS caused by wall motion is expected to be dominantly axial, and this was confirmed by creating polar plots of instantaneous WSS vectors over the cardiac cycle for regions in proximal and distal parts of the vessel (Supplementary Figure S7). The magnitude of the vectors increased between proximal and distal parts of the vessel, consistent with the trend in Supplementary Figure S6. The total forward wall displacement should equal the total backward displacement at any particular location since the net deformation of the vessel over a complete cardiac cycle is zero at all locations. WSS generated by this displacement is expected to follow the same trend and indeed the polar plots do show roughly equal excursions in the forward and backward direction. This had two consequences: the mean WSS vector was very low (on the order of 10^–2^ Pa), and values of the OSI were everywhere close to its theoretical maximum of 0.5 (Supplementary Figure S6).

CFI and transWSS for the zero-inflow condition are indeterminate – the mean WSS vector, which is required for their calculation, is theoretically 0,0 when flow is purely oscillatory, and is small in magnitude and arbitrary in direction when flow is nearly so (Peiffer et al., 2013). To estimate the contribution of wall motion to these shear metrics, and to TAWSS and OSI, we subtracted the WSS vectors for the zero-inflow case from the vectors for the case of dynamic geometry with pulsatile flow. Maps of the recomputed metrics are shown in Supplementary Figure S8 and means for the entire maps are given in Supplementary Table S3. Decreases in all metrics are discernible when the zero-inflow vectors are subtracted from the physiological case (*cf* dynamic geometry with pulsatile inflow sub-parts of Figure 4), but only on close inspection; the overall trends are unchanged.

## Limitations

The RCA does not have major bifurcations, unlike the left coronary artery system, but it does have smaller branches. These were not captured in the model since the diameter of the branch arteries is smaller than the MRI slice thickness. This means that the loss of blood to these branches is not included in the model and hence the mean flow rate will be increasingly too high on progressing down the vessel. TAWSS and transWSS magnitude will show the same trend, and hence are not suitable at this stage for comparison with, say, patterns of lesions; the data show only how these metrics are affected locally by coronary motion and blood viscosity. An estimate of the size of this effect was obtained by removing taper from the RCA, a method previously employed by Zeng et al. (Zeng et al., 2003) which assumes that mean axial velocity is constant at all points along the vessel. Substantial changes are seen (Supplementary Figure S9): in particular, the largest values of the dimensional TAWSS and transWSS metrics occur in the proximal rather than distal parts of the vessel. Smaller changes occur in the non-dimensional OSI and CFI metrics; these must reflect changes in flow structure as well as rate.

The subject-specific waveform, obtained by phase-contrast MRI at the proximal end of the vessel, indicates that cross-sectionally averaged blood flow was in the proximal-to-distal direction at all times during the cardiac cycle. Some published waveforms show periods of reverse flow at other locations. If such a waveform were imposed, it would at least have the effect of increasing OSI values, even in the absence of secondary flow.

Future work should incorporate branches, add geometries from other subjects, include a parametric study of inflow waveforms, and compare patterns of WSS metrics with the pattern of disease.

## Conclusion

This study confirms previous observations that the TAWSS is not substantially affected by coronary artery motion, and that the OSI is affected more. A novel observation is that metrics capturing transverse flows – the CFI and transWSS – are affected even more than the OSI; this effect can be attributed to changing vortical structures resulting from the dynamic curvature. An additional finding was that models of non-Newtonian blood rheology can also have a significant influence if they increase viscosity at most locations and times; that leads to increased magnitude of dimensional shear metrics but also to changes in the relative magnitudes of shear metrics from location to location. Hence studies of transWSS in coronary arteries should take vessel motion into account and, depending on the characteristics of the subject, might also need to account for non-Newtonian blood rheology. This is necessary for valid comparisons with the distribution of coronary disease.

## Data Availability

The raw data supporting the conclusion of this article will be made available by the authors, without undue reservation.
