# Supplementary material for: Influence of right coronary artery motion, flow pulsatility and non-Newtonian rheology on wall shear stress metrics
Source: Front Bioeng Biotechnol. 2022 Aug 9;10:962687. doi: 10.3389/fbioe.2022.962687 (PMC9395597; doi:10.3389/fbioe.2022.962687)
Supplement: Supplementary file 1 [file DataSheet1.docx]

**SUPPLEMENTARY MATERIAL**


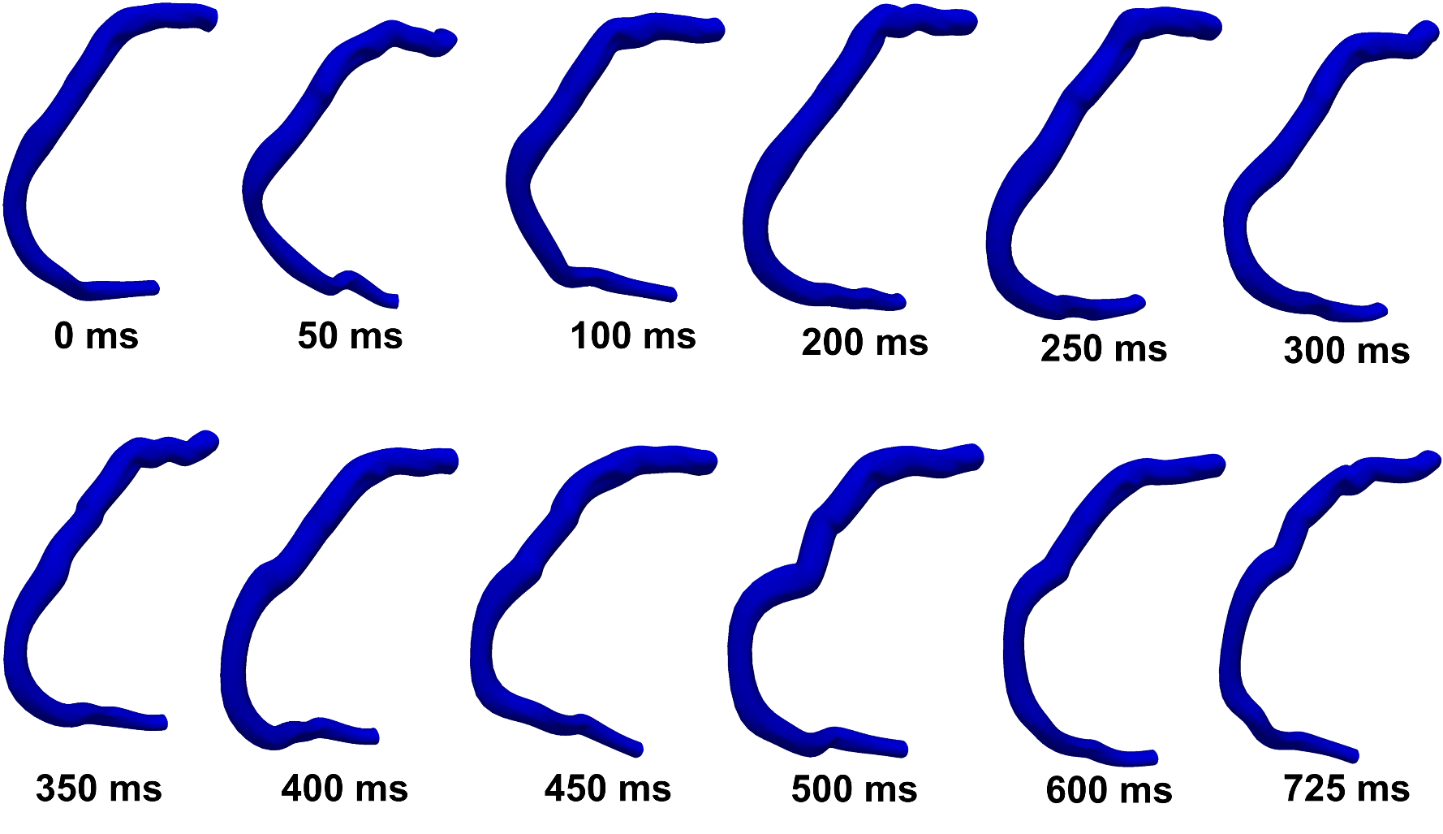


Supplementary Figure S1. Geometry of an RCA at various time points in the cardiac cycle


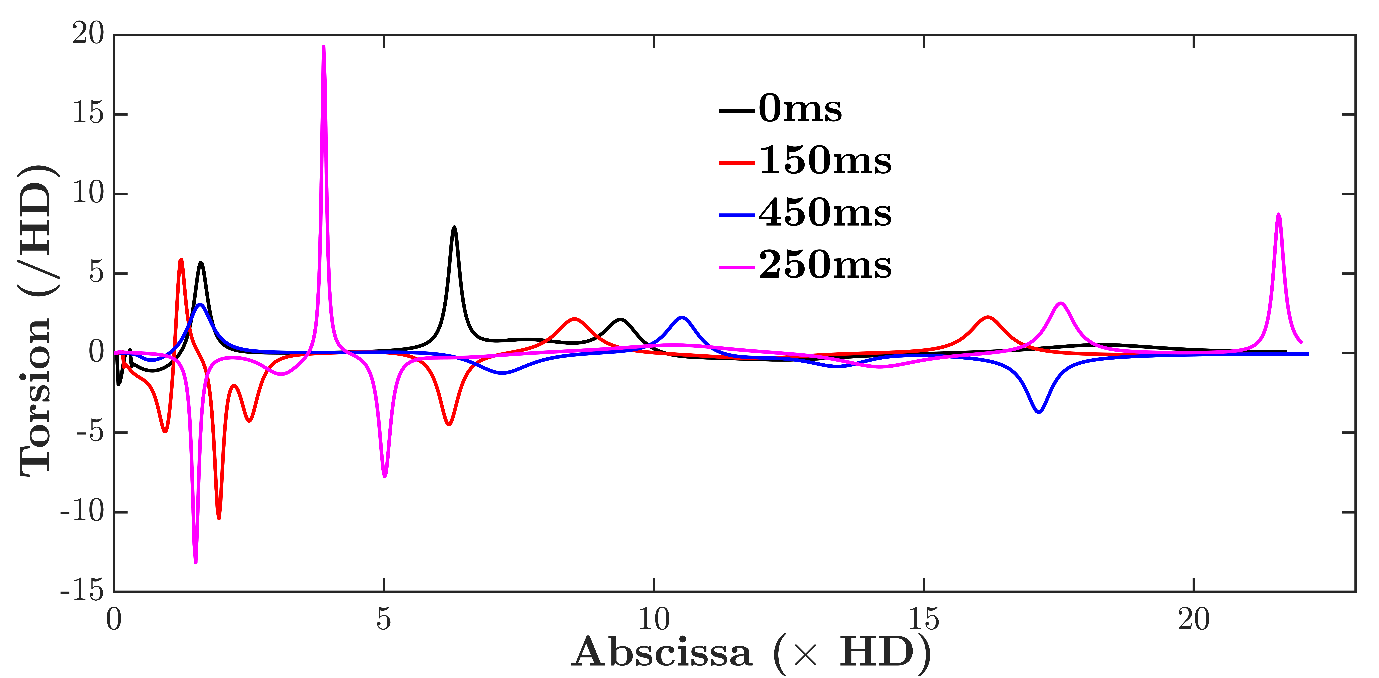

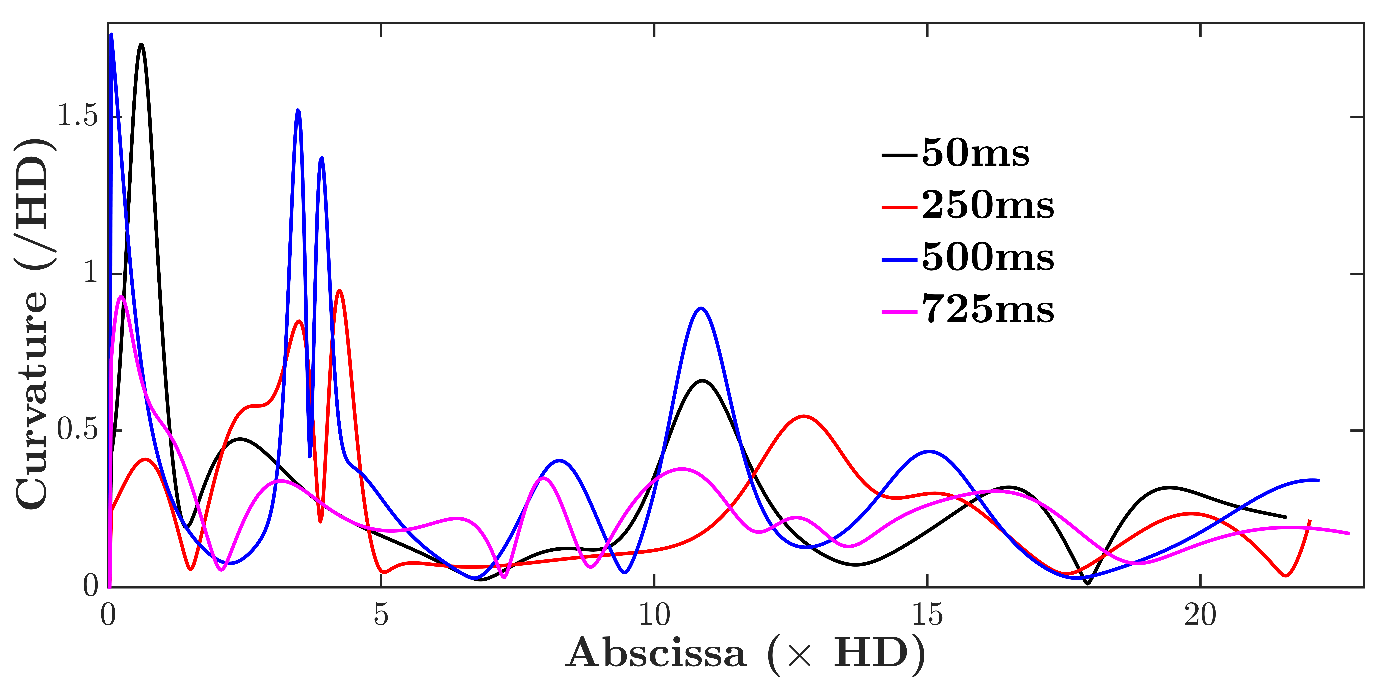


Supplementary Figure S2. Curvature (top) and torsion (bottom) along the length of the RCA at various time points in the cardiac cycle.


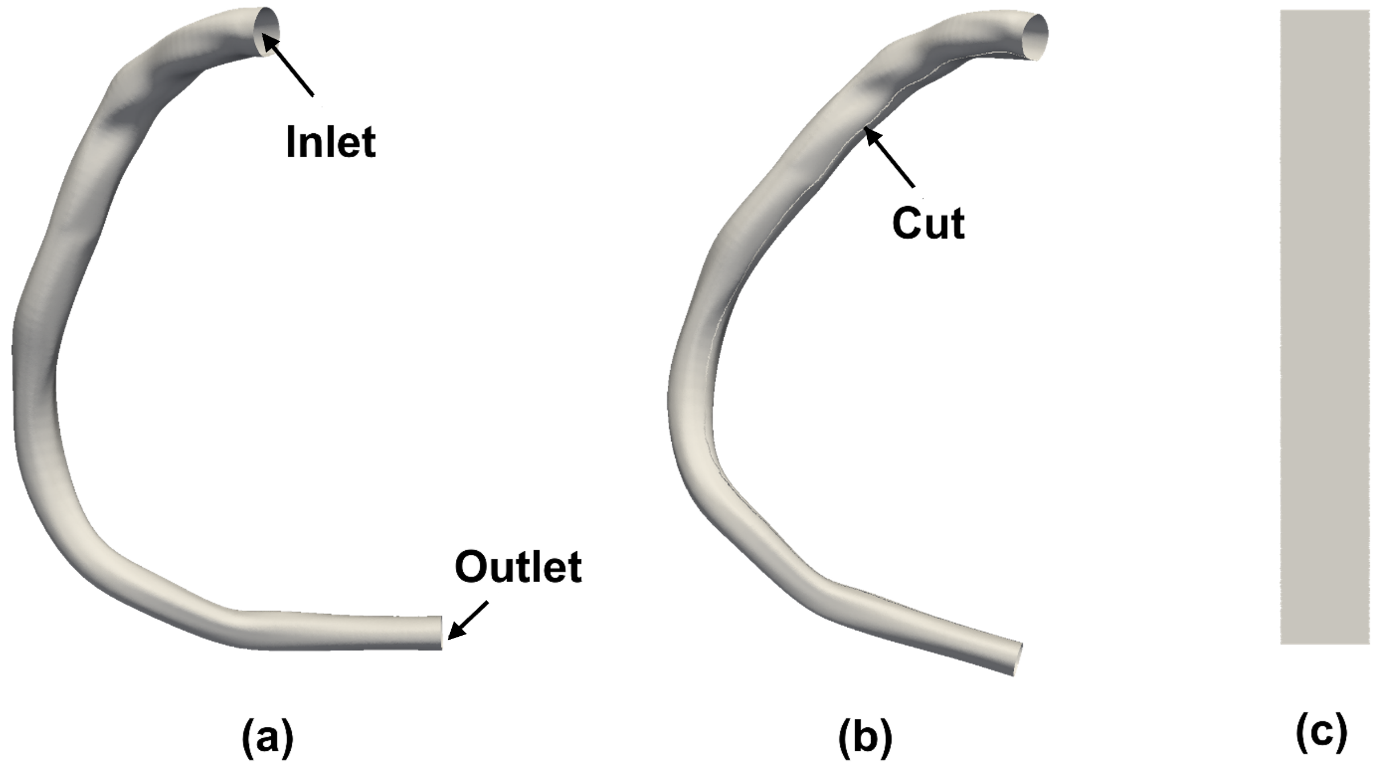


Supplementary Figure S3: Unwrapping 3-D RCA geometry at t=0 into a rectangular surface. (a) 3-D geometry at t=0, (b) 3-D geometry cut open along the inner curvature of the vessel and (c) 3-D geometry unfolded into rectangular surface


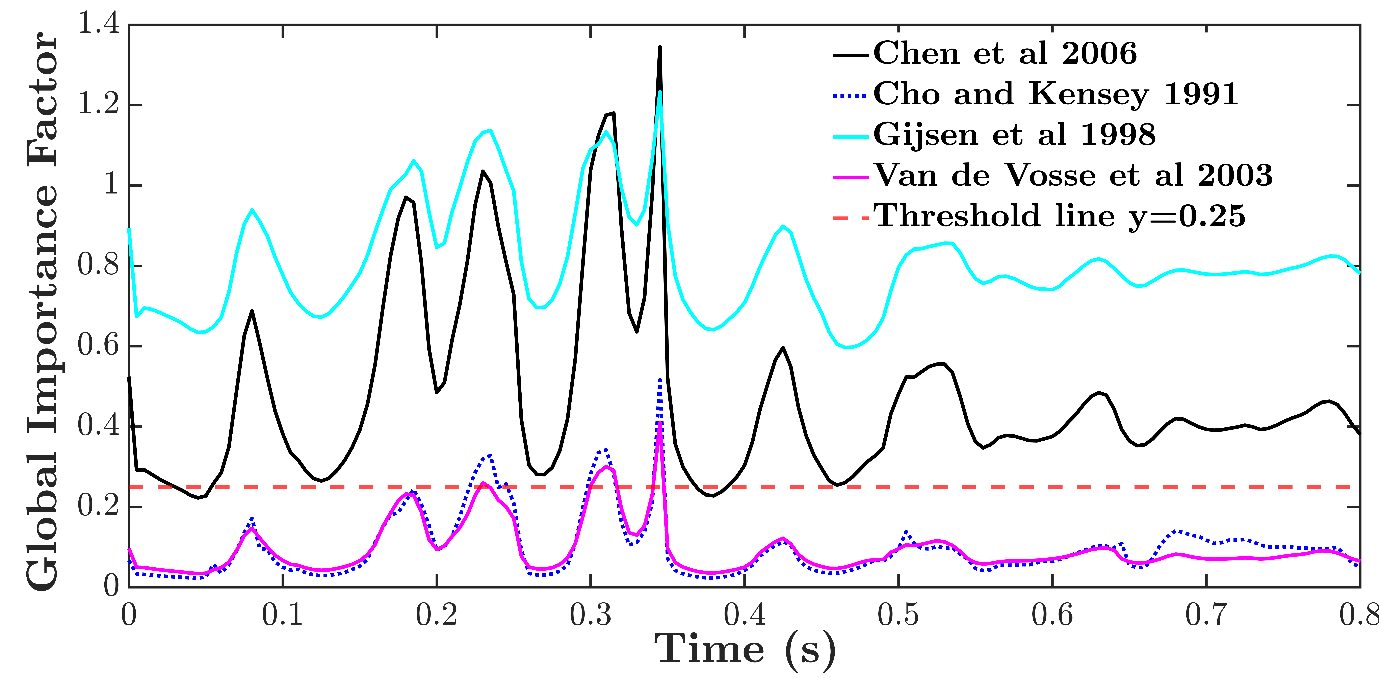


Supplementary Figure S4. Global importance factor for four Carreau-Yasuda non-Newtonian models


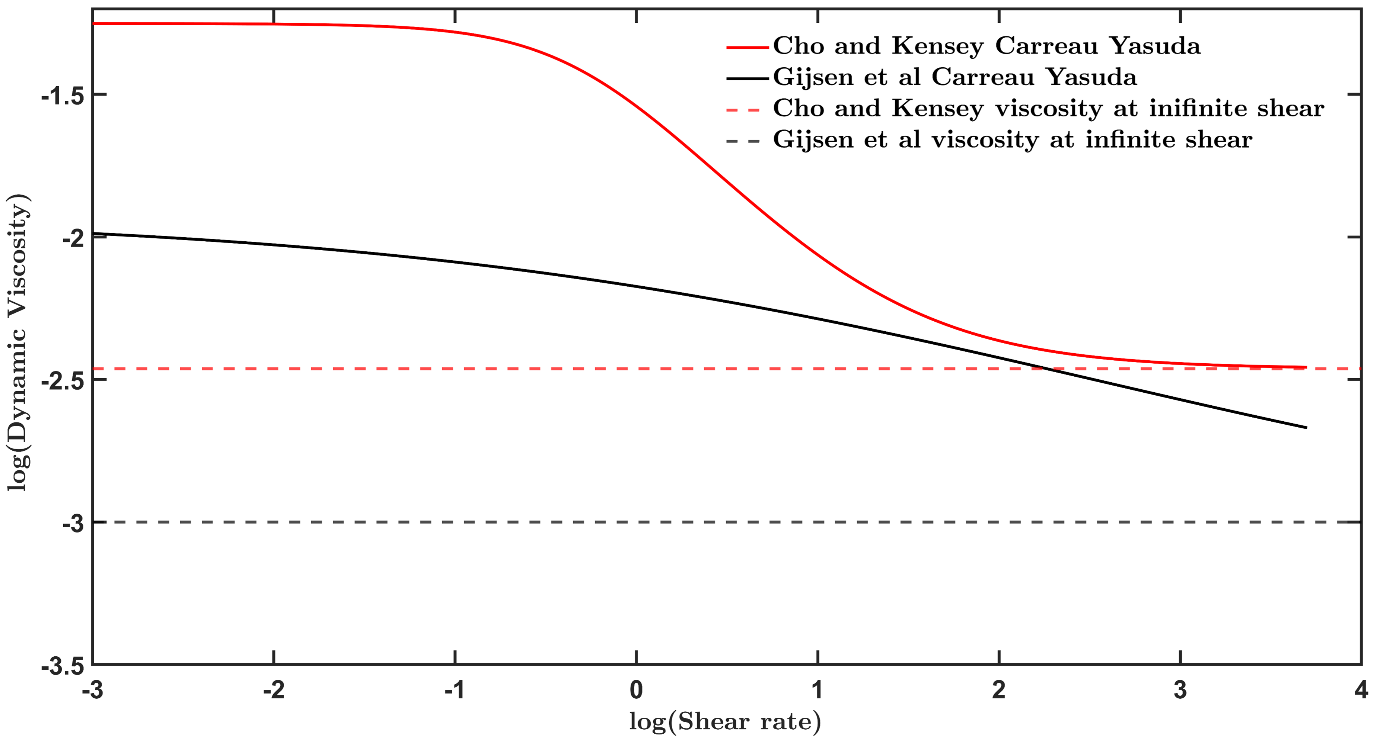


Supplementary Figure S5. Dynamic viscosity versus shear rate for the models of Cho and Kensey (1991) and Gijsen et al. (1998).


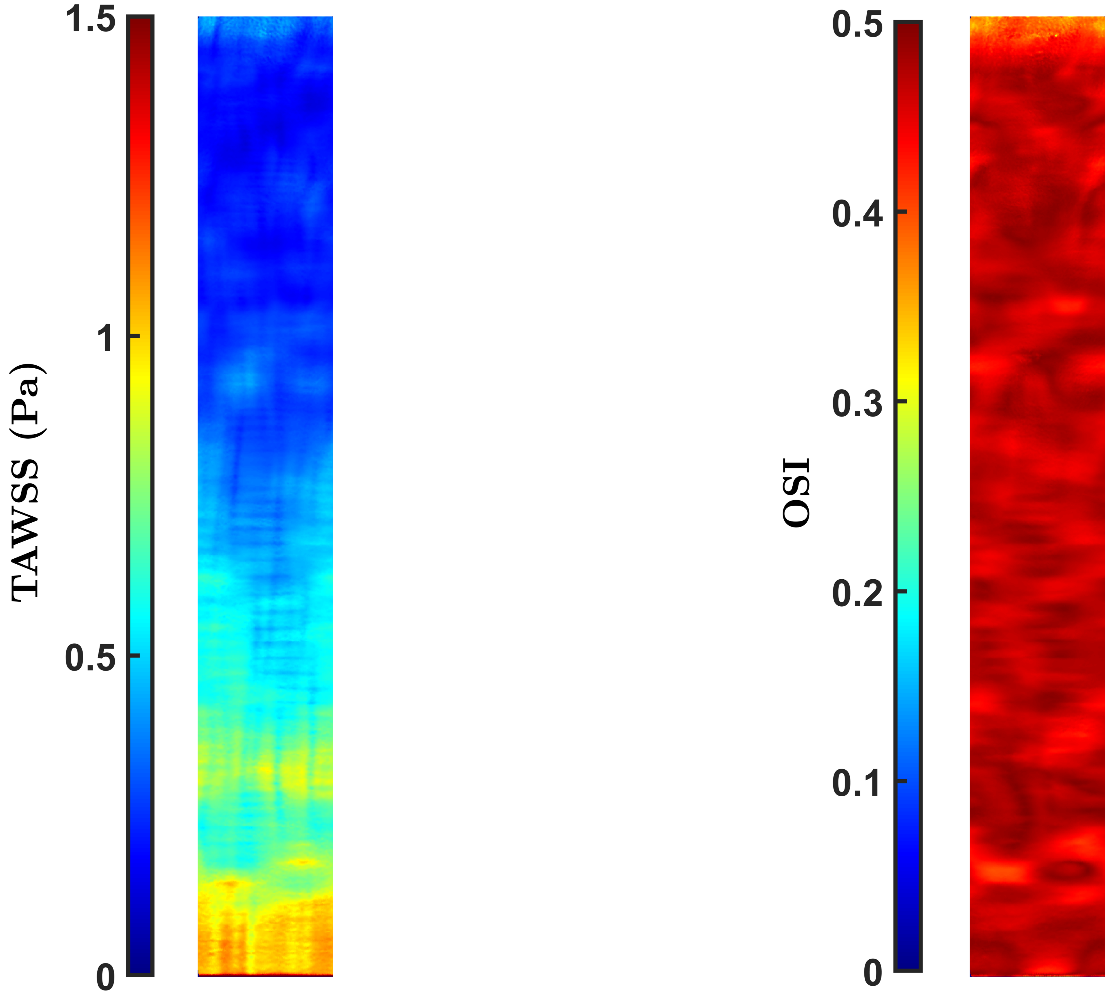


Supplementary Figure S6: Maps of TAWSS (left) and OSI (right) for the zero inflow, dynamic geometry case.


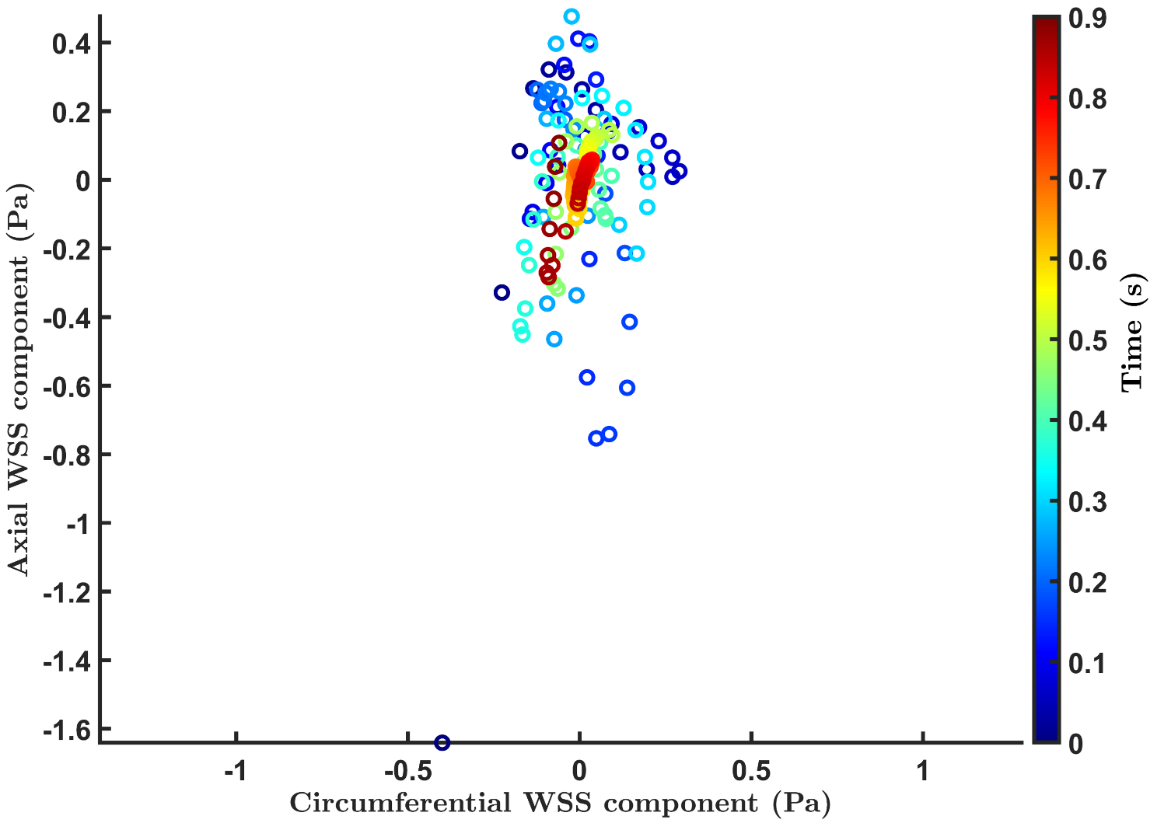

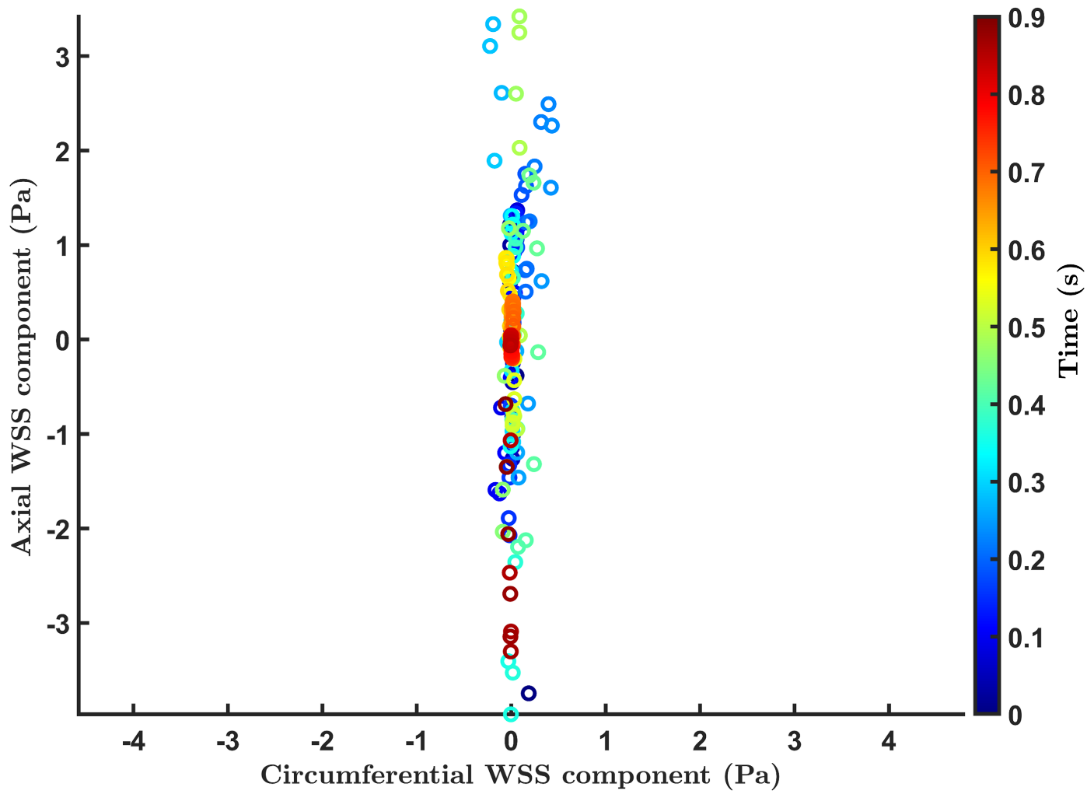


Supplementary Figure S7. Polar plots of instantaneous WSS vectors throughout the cardiac cycle in the zero-inflow dynamic geometry case for proximal (top) and distal (bottom) regions of the RCA. Note the different scales.


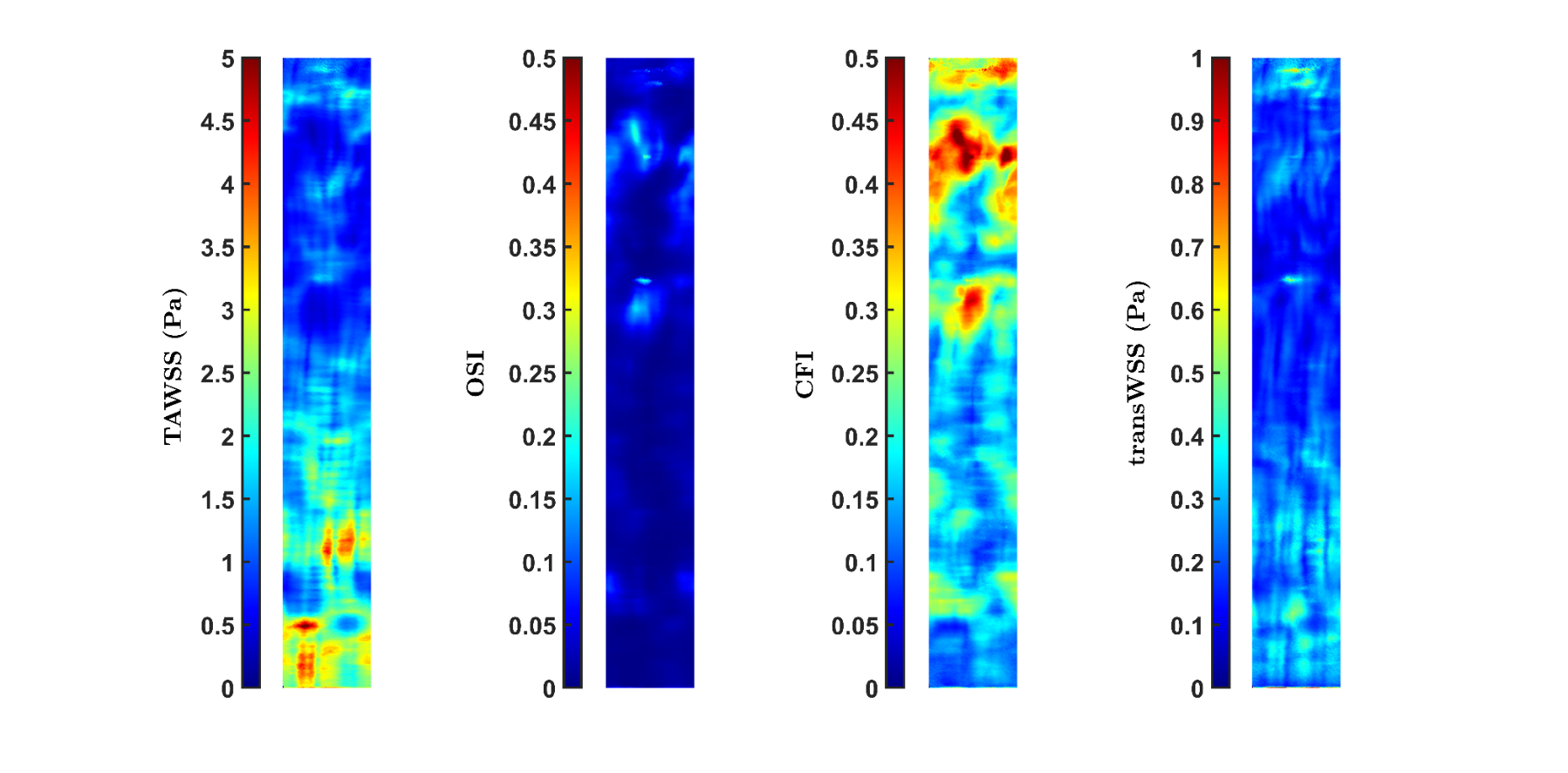


Supplementary Figure S8. Maps of TAWSS, OSI, CFI and transWSS for the physiological case (pulsatile inflow, dynamic geometry sub-parts of Figure 4) but with the WSS vectors for the zero-inflow, dynamic geometry case subtracted.


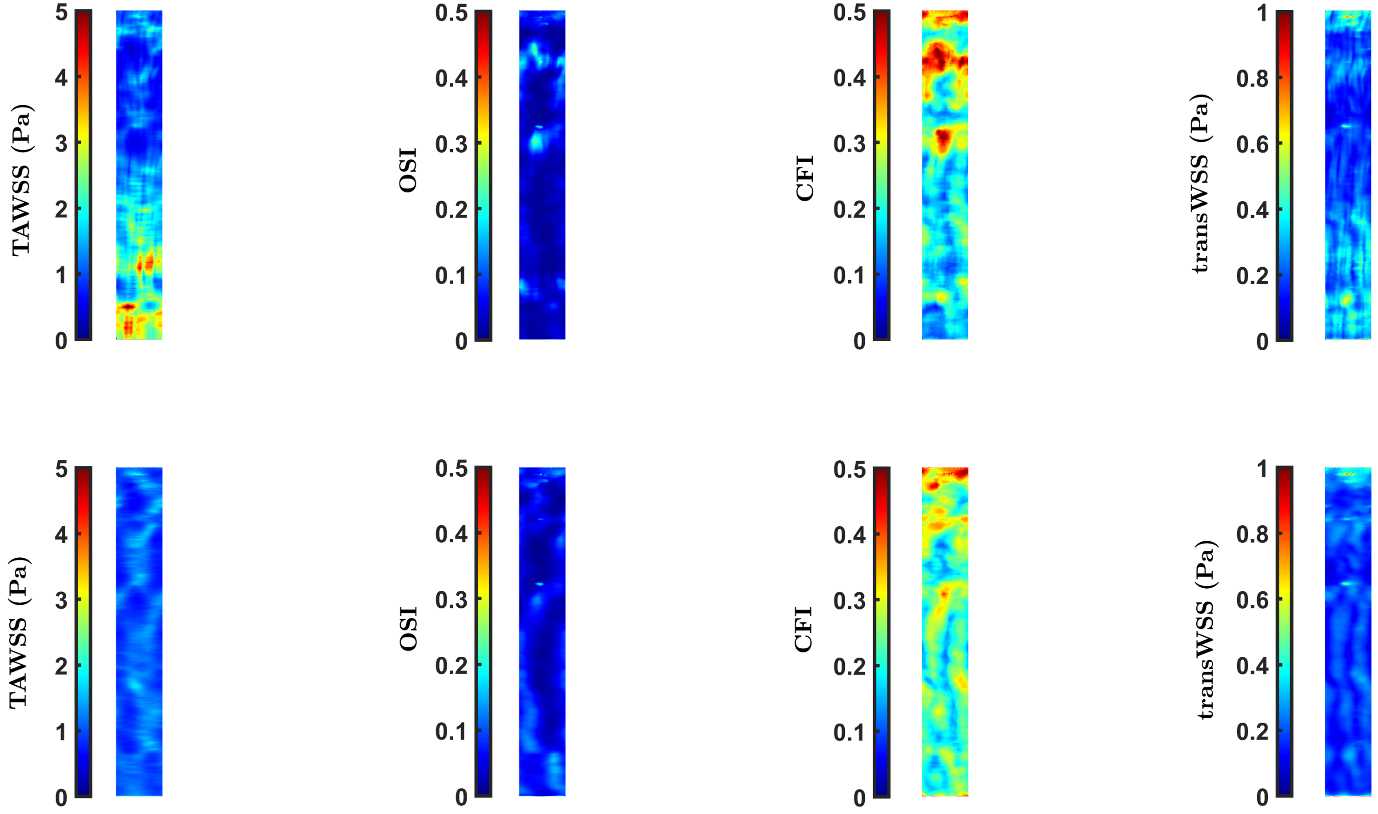


Supplementary Figure S9. Comparing the four metrics for tapered (top) and non-tapered (bottom) RCA geometries.

Supplementary Table S1: Carreau-Yasuda parameters from four different studies

|  |  | **Parameters** | | | |
| --- | --- | --- | --- | --- | --- |
|  | $\boldsymbol{\mu}_{\boldsymbol{\infty}}$ **(Pa s)** | $\boldsymbol{\mu}_{\boldsymbol{0}}$ **(Pa s)** | **a** | **n** | $\boldsymbol{\lambda}$ **(s)** |
|  |  |  |  |  |  |
| **Cho and Kensey (1991)** | 0.00345 | 0.056 | 1.25 | 0.22 | 1.902 |
| **Gijsen et al. (1998)** | 0.001 | 0.012 | 0.222 | 0.681 | 0.086 |
| **Van de Vosse et al. (2003)** | 0.00476 | 0.0519 | 0.409 | 0.191 | 0.438 |
| **Chen et al. (2006)** | 0.0022 | 0.022 | 0.644 | 0.392 | 0.11 |

Supplementary Table S2: Spearman rank correlation coefficients for maps of instantaneous WSS under the three conditions, shown for six time points during the cardiac cycle. ES, PS and PD indicate early systole, peak systole and peak diastole, respectively.

**t=0.05 s t=0.1 s t=0.3 s t=0.4 s t=0.6 s t=0.725 s**

**(ES) (PS) (PD)**

**Static Pulsatile**

**vs Dynamic Pulsatile** 0.81 0.82 0.81 0.86 0.79 0.83

**Dynamic Nonpulsatile**

**vs Dynamic Pulsatile** 0.99 0.99 0.98 0.99 0.98 0.99

Supplementary Table S3: Mean values of WSS metrics for the physiological case (pulsatile inflow, dynamic geometry) recomputed after vectors for the zero inflow, dynamic geometry case had been subtracted.

**Mean values of: TAWSS (Pa) OSI CFI transWSS (Pa)**

**Dynamic pulsatile case** 1.47 0.046 0.235 0.232

**Dynamic pulsatile case**

**minus zero inflow case**  1.41 0.029 0.223 0.221
